# Effect of combined music and touch intervention on pain response and β-endorphin and cortisol concentrations in late preterm infants

**DOI:** 10.1186/s12887-016-0755-y

**Published:** 2017-01-26

**Authors:** Jie Qiu, Yun-fei Jiang, Fang Li, Qian-hong Tong, Hui Rong, Rui Cheng

**Affiliations:** 1 Department of Newborn Infants, Children’s Hospital of Nanjing Medical University, Nanjing, 210008 China; 2 Department of Pediatrics, Yixing People’s Hospital Affiliated to Jiangsu University, Yixing, 214200 China

**Keywords:** Preterm infant, Pain, Cortisol, β-Endorphin, Music, Touch

## Abstract

**Background:**

Preterm neonates undergo many painful procedures as part of their standard care in the neonatal intensive care unit. However, pain treatment is inadequate in many of these routine procedures. In the present study, we investigated the impact and mechanism of combined music and touch intervention (CMT) on the pain response in premature infants.

**Methods:**

Sixty-two preterm neonates (gestational age of <37 weeks) were randomly assigned to either the experimental or control group. Infants in the experimental group underwent painful procedures with CMT, and those in the control group underwent painful procedures without CMT. Blood samples were collected from all infants at the beginning of hospitalization and 2 weeks later to assess the cortisol and β-endorphin concentrations. Differences in the levels of cortisol and β-endorphin between two groups were examined using analysis of covariance (ANCOVA).

**Results:**

In total, 3707 painful procedures were performed on 62 neonates during their hospitalization. The average number of painful procedures in the control group (*n* = 35.5) was higher than that in the experimental group (*n* = 29.0) during hospitalization, although no significant difference was reached (*P* > 0.05). After 2 weeks, the Premature Infant Pain Profile scores were significantly higher in the control group than experimental group (13.000 ± 0.461 *vs* 10.500 ± 0.850, respectively; *P* < 0.05). The cortisol concentration was not significantly different between the control and experimental groups either at the beginning of hospitalization (131.000 ± 18.190 *vs* 237.200 ± 43.860, respectively; *P* > 0.05) or 2 weeks later (162.400 ± 23.580 *vs* 184.600 ± 21.170, respectively; *P* > 0.05). However, the serum β-endorphin concentration was higher in the experimental group than in the control group both at the beginning of hospitalization (1.640 ± 0.390 *vs* 1.179 ± 0.090, respectively; *P* < 0.05) and 2 weeks later (2.290 ± 0.740 *vs* 1.390 ± 0.410, respectively; *P* < 0.05).

**Conclusions:**

CMT might decrease the pain response of preterm neonates by significantly improving the β-endorphin concentration, but not the blood cortisol concentration.

**Trial registration:**

Current Controlled Trials ISRCTN14131492. Registered on 01 Aug 2016.

## Background

Preterm neonates undergo many painful procedures as part of their standard care in the neonatal intensive care unit (NICU) [1, 2]. Accumulating evidence shows that preterm infants are able to experience pain [3, 4] and are highly sensitive to pain because of their immature and vulnerable nervous systems [5]. Repetitive, prolonged, and poorly treated pain has many deleterious consequences. Short-term effects include excessive crying, choking, gagging, vomiting, and long-term effects include altered pain sensitivity as well as permanent neuroanatomical and behavioral abnormalities [6]. Therefore, there is an urgent need to establish safe and effective treatments for pain relief in infants.

However, pain treatment for routine procedures in the NICU is inadequate. In fact, many doctors are reluctant to use analgesic medications such as nonsteroidal anti-inflammatories or acetaminophen in the NICU because the effectiveness of these medications has not been proven or because of potential adverse effects in the short term (e.g., opioid-induced ileus or apnea) or long term (e.g., ketamine-induced neuroapoptosis). Various nonpharmacological treatments, including non-nutritive sucking both with and without sucrose, swaddling or kangaroo care, music therapy, and multisensorial stimulation, reportedly exert a pain-modulating effect on preterm neonates because they activate the neonates’ attention, distract them from the pain, and thus modify pain perception [7–14]. Music therapy may help to relieve procedural pain in both full-term and preterm infants because it can provide an auditory stimulus that modulates pain perception, obviating or decreasing the need for pharmacological agents [10, 12, 13, 15, 16]. Another nonpharmacological intervention, touching, also helps to reduce pain in preterm and term neonates. Prasopkittikun and Tilokskulchai [17] reported that touching, swaddling, maternal holding, and repositioning were effective nonpharmacological interventions that reduced pain using validated pain assessment measures in preterm and term infants. However, a combination of these nonpharmacological interventions may be more useful because their effectiveness may vary across infants. Furthermore, few data specifically address premature infants, who are exposed to the most procedural pain and are the most vulnerable to altered developmental trajectories in childhood. Especially in China, no study has addressed the effects of combined music and touch intervention (CMT) on the pain response in late preterm infants. Therefore, in the present study, we investigated the impact of CMT on the premature infant pain response. We also studied the levels of cortisol and β-endorphin after the intervention because these parameters may provide evidence of the effectiveness of CMT in alleviating pain in premature infants. Pain may activate the hypothalamic-pituitary-adrenal (HPA) axis, inducing an endocrine response in premature infants that increases cortisol levels [18]. Cortisol affects the metabolism, cardiovascular system, and central nervous system [19]. In various studies, cortisol has been used to assess the effects of nonpharmacological interventions against pain in newborn infants, including sucrose, the kangaroo position, and developmental care [20–22]. β-Endorphin is an endogenous opioid that is released in response to pain and increases the inhibition of pain at several sites within the β-endorphin inhibitory pathway. It is released when an organism is exposed to stress or painful stimuli. During acute stress and pain, the HPA system increases the blood β-endorphin concentration. This circulatory release of β-endorphin can be reduced by systemic analgesia [23–25]; thus, plasma β-endorphin concentrations have frequently been used to determine analgesic efficacy.

The purpose of this study was to examine whether CMT is an effective pain management method for premature infants during the painful procedures performed on a daily basis in the NICU. We compared the Premature Infant Pain Profile (PIPP) scores and cortisol and β-endorphin concentrations between infants who did and did not receive CMT. To the best of our knowledge, this is the first study to examine the relationship between CMT and the pain response in premature infants.

## Methods

### Participants

This randomized controlled trial was conducted in the NICU of Children’s Hospital of Nanjing Medical University from June 2011 to March 2012. The inclusion criteria were as follows: (1) admission within 72 h after birth and (2) a gestational age of <37 weeks. The exclusion criteria were one or any combination of the following: (1) serious birth injuries, (2) serious malformations (especially in the oral cavity or external ear), (3) significant parenchymal brain injury (grade IV intraventricular hemorrhage or periventricular leukomalacia), (4) treatment with analgesics or sedatives within 72 h of the assessment, or (5) failed hearing screening.

### Procedures

The research team comprised a neonatologist, three research nurses, a child health care expert, and three assistants with extensive research and clinical experience. A list of daily painful procedures was designed [26–28]; this list also included other procedures that the clinicians or nurses considered painful, such as tracheal aspiration, nasal aspiration, removal of intravenous lines, and removal of adhesives (Table 2). After consent was obtained from the infants’ parents, the infants were randomly assigned to either the experimental or control group using a random numbers table. Infants in the control group underwent daily painful procedures without intervention. Infants in the experimental group underwent painful procedures with CMT. Only nurses on the research team administered the CMT to ensure consistency. A compact disc player (AZ-1103; Philips, Amsterdam, Netherlands) was used for the music intervention. Audio stimulation was provided by “Smart Baby Lullaby” compact discs. The music included lullabies and nursery rhymes, which are musically simple songs with a lower pitch and slower tempo. The most appropriate decibel level for the disc player was determined to be 55 to 65 decibels (dB) using an A-weighted scale, measured using a TES-1351B Sound Level Meter (TES Electrical Electronic Corp., Taipei, Taiwan). This volume level was implemented to meet criteria consistent with current knowledge of infant auditory development in relationship to the ambient NICU sound environment. Music applications of 55 to 65 dB for short-timed interventions met the recommendations for infants and supported a variance of 10 dB between the ambient sound floor and sound interventions to ensure audibility [29]. Although environmental sound was not within the scope of this investigation, the staff members in our NICU were cognizant of the noise and ambient sounds. The disc player was placed approximately 15 to 20 cm above the infants’ heads, which allowed for continuous play of the music from 5 min before the experimental procedure until 30 min after the procedure. The touch intervention protocol (Gentle Human Touch, GHT), started from the beginning of each procedure until 10 min after the procedure and was performed as previously described [30]. Briefly, the nurse gently placed her left hand on the infant’s head with her fingertips resting immediately above the eyebrow line and her palm touching the infant’s crown. Her right hand was placed with the right thumb on the infant’s right shoulder (midline position) with the rest of her hand and fingers on the infant’s arm, above the elbow. The video camera was positioned for a close-up of the face. The signals were fed directly to a VCR before, during, and after each painful procedure. These procedures were repeated for every painful procedure during the 2-week data collection period.

### PIPP

The PIPP is a pain measure for premature infants and comprises seven indicators: two contextual (gestational age and behavioral state), two physiologic (heart rate and oxygen saturation), and three behavioral (brow bulge, eye squeeze, and nasolabial furrow). Each indicator is scored on a 4-point scale (0–3) for a maximum total score of 21 [31, 32]. The internal consistency and inter- and intra-rater reliability were summarized with good face validity and content validity in a review article on pain assessment in children [32]. Video sequences were assessed by three different nurses. The three nurses were supervised to ensure that they did not consult one another, and all were blinded to the intervention *vs* control group.

### Cortisol and β-endorphin concentrations

The β-endorphin concentration is known to exhibit a circadian rhythm that repeats once in a 24-h period. Therefore, a 2-ml blood sample was collected from each infant between 6 and 7 AM to minimize any circadian rhythm effects. The cortisol and β-endorphin concentrations were quantitatively assessed using a highly sensitive electrochemiluminescence immunoassay (Roche Diagnostics, Mannheim, Germany) and a modular analytics analyzer (Elecsys Modular Analytics E170; Roche Diagnostics, Tokyo, Japan).

### Statistical analysis

Unless otherwise stated, numerical results are presented as the mean ± standard error of the mean. Statistical analyses were performed using the SPSS 19.0 statistical package (IBM Corp., Armonk, NY, USA). Demographic data and pain experience were compared between the two groups using the unpaired *t*-test (normally distributed data), the Mann–Whitney U test (non-normally distributed data), or Fisher’s exact test (categorical data). Gestational ages were compared using one-way analysis of variance and the least significant difference test. Wilcoxon signed-rank tests were carried out to examine PIPP scores. ANCOVA was used to evaluate the difference in the levels of cortisol and β-endorphin between two groups. Statistical significance was defined as *P* < 0.05.

## Results

### Demographic variables

**Fig. 1:**
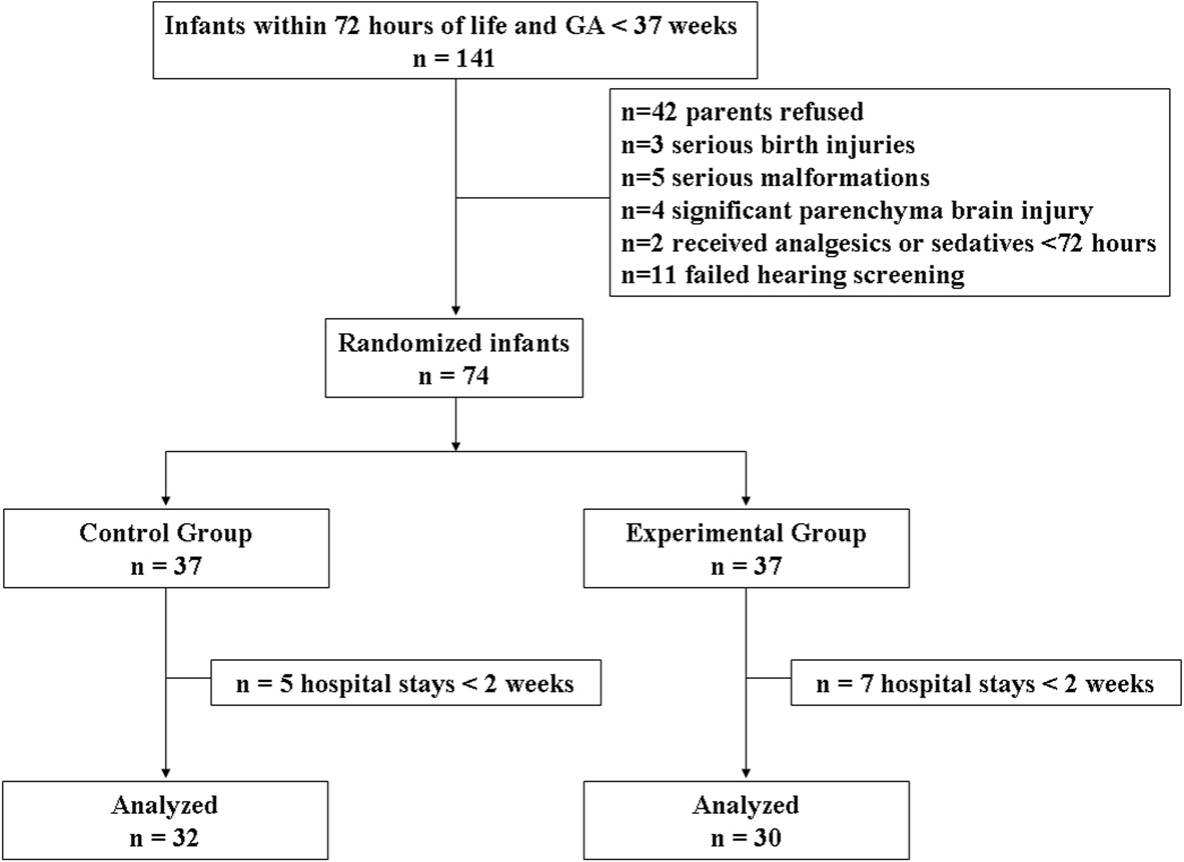
Study profile and patient flow

During the study period (June 2011 to March 2012), 74 of 141 eligible infants were enrolled in this study (flow diagram, Fig. 1). We excluded seven neonates in the experimental group and five neonates in the control group who were discharged within 2 weeks. Thus, 62 neonates were finally included in the analysis. The primary purpose of the pilot study was to refine the study methodology and obtain adequate data with which to conduct an analysis for an expanded study. Based on the advice of a biostatistician, a sample size of 30 was determined to be adequate for this purpose. In addition, the severity of illness was assessed using the Score for Neonatal Acute Physiology II [33]. Table 1 shows the demographic characteristics of the neonates. There were no differences in the demographic characteristics between the experimental and control groups.

**Table 1:** Clinical and demographic characteristics of neonates

	Control group *n* = 32	Experimental group *n =* 30	*P*
Gestational age (weeks)	33.33 ± 0.54	34.30 ± 0.67	0.10
Birth weight (kg)	2.00 ± 0.07	1.93 ± 0.13	0.64
Sex (male/female)	15/17	13/17	0.80
1-min APGAR	8.67 ± 0.24	9.37 ± 0.32	0.96
5-min APGAR	9.40 ± 0.22	9.64 ± 0.15	0.47
Severity (SNAP-II)	8 (0–57)	8 (0–33)	0.69

Data are presented as mean ± SD or median (range)

APGAR indicates appearance, pulse, grimace, activity, respiration

### Numbers and types of pain experiences

In total, 3707 painful procedures were performed on 62 neonates during their hospitalization. Among these procedures, 1913 were performed in the control group and 1794 were performed in the experimental group. The median number of painful procedures for each preterm neonate in the control group was 35.5 (range, 18–325). The median number in the experimental group was 29 (range, 14–316). The average number of painful procedures in the control group was higher than that in the experimental group during hospitalization, although no statistical difference was reached (*P* > 0.05). The types of painful procedures performed on the neonates are listed in Table 2.

**Table 2:** Types of painful procedures on neonates

Procedure Types	Procedure no. (%) on control group	Procedure no. (%) on experimanetal group
Tracheal aspiration	183 (9.57)	165 (9.20)
Nasal aspiration	283 (14.79)	241 (13.43)
Intravenous cannulation	256 (13.38)	231 (12.88)
Removal of intravenous lines	229 (11.97)	206 (11.48)
Adhesive removal	229 (11.97)	206 (11.48)
Fingerstick	89 (4.65)	95 (5.30)
Heelstick	69 (3.61)	85 (4.74)
Femoral venous puncture	108 (5.65)	107 (5.96)
Arterial puncture	53 (2.77)	49 (2.73)
Laxative or enema	48 (2.51)	43 (2.40)
Gastric tube insertion	34 (1.78)	30 (1.67)
Tracheal intubation	7 (0.37)	6 (0.33)
Tracheal extubation	7 (0.37)	6 (0.33)
Chest physiotherapy	285 (14.90)	295 (16.44)
Lumbar puncture	9 (0.47)	8 (0.45)
Intradermal injection	24 (1.25)	21 (1.17)
Total	1913 (100.01)	1794 (99.99)

### PIPP

**Fig. 2:**
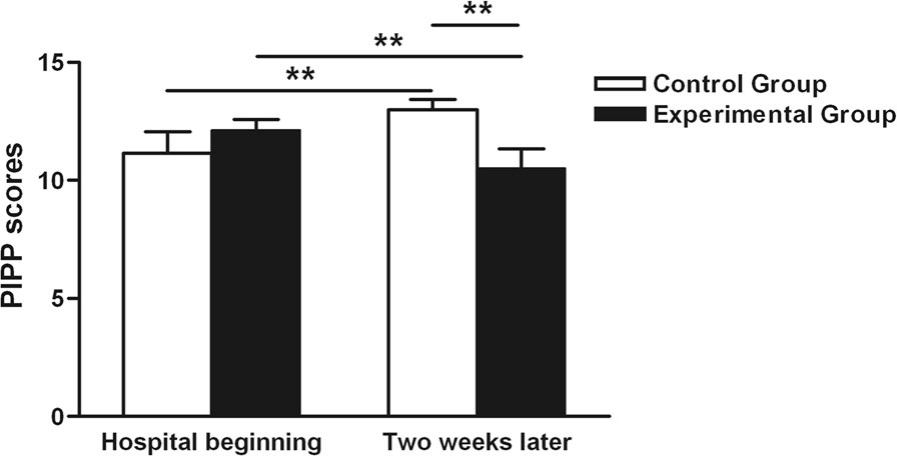
Effects of CMT on PIPP. The mean PIPP scores were 11.17 ± 0.91 and 12.14 ± 0.46 (control vs. experimental) at the beginning of the hospitalization, respectively. There was no significant difference between the two groups at the beginning of hospitalization (**P > 0.05). Two weeks later, the experimental group had significantly lower scores than the control group (10.50 ± 0.85 *vs* 13.00 ± 0.46, **P < 0.05)

The mean PIPP scores were 11.17 ± 0.91 and 12.14 ± 0.46 (control *vs*. experimental), respectively, at the beginning of the hospitalization. After 2 weeks, the PIPP score in the control group had significantly increased (t = 2.573; *P* < 0.05) and that in the experimental group had significantly decreased (t = 2.216; *P* < 0.05). There was no significant difference between the two groups at the beginning. Two weeks later, however, the experimental group had a significantly lower score than the control group (10.50 ± 0.85 *vs* 13.00 ± 0.46, respectively; *P* < 0.05) (Fig. 2).

### Cortisol concentration

**Fig. 3:**
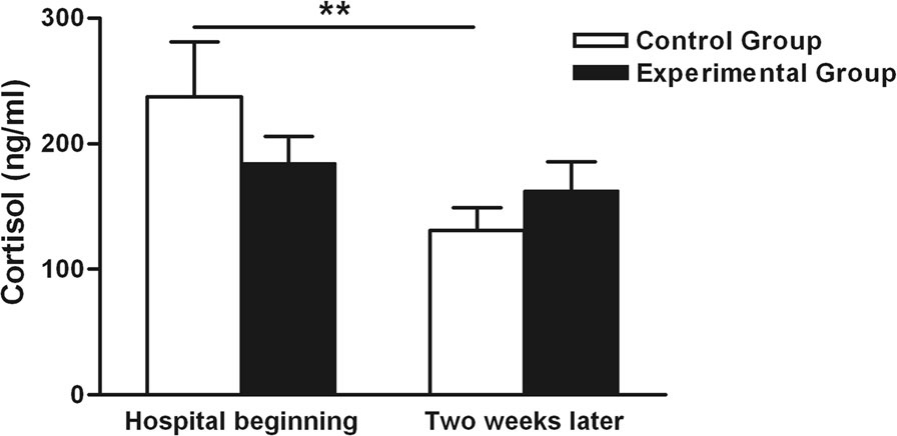
Effects of CMT on cortisol concentration. In the control group, the cortisol concentration had significantly decreased after 2 weeks of hospitalization *vs* the beginning (131.00 ± 18.19 *vs* 237.20 ± 43.86, respectively; **P < 0.05), while there was no significant change in the experimental group after 2 weeks *vs* the beginning (162.40 ± 23.58 *vs* 184.60 ± 21.17, respectively; **P > 0.05). No significant difference was noted between the two groups either at the beginning of hospitalization or 2 weeks later

In the control group, the cortisol concentration significantly decreased after 2 weeks of hospitalization (131.00 ± 18.19 *vs* 237.20 ± 43.86, respectively; *P* < 0.05). Additionally, no significant changes occurred in the experimental group from the beginning of hospitalization to 2 weeks later (184.60 ± 21.17 *vs* 162.40 ± 23.58, respectively; *P* > 0.05). No significant differences were noted between the two groups either at the beginning of hospitalization or 2 weeks later (Fig. 3).

### β-Endorphin

**Fig. 4:**
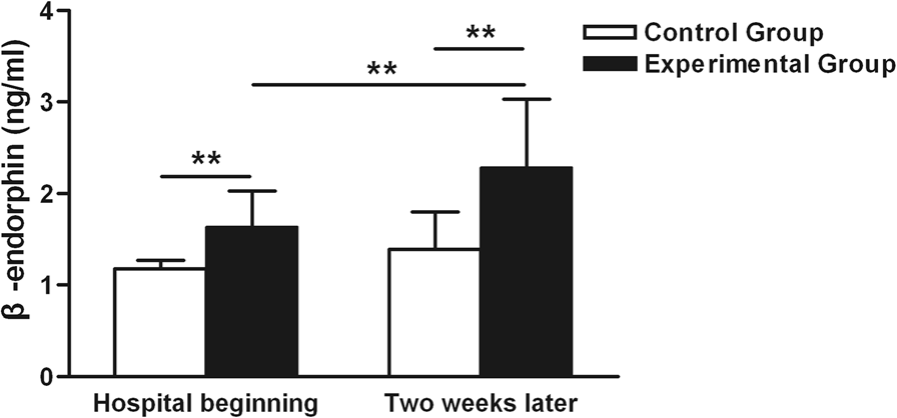
Effects of CMT on β-endorphin concentration. Neonates in the experimental group had higher serum β-endorphin levels than those in the control group both at the beginning of hospitalization (1.64 ± 0.39 *vs* 1.18 ± 0.09, respectively; **P < 0.05) and 2 weeks later (2.29 ± 0.74 *vs* 1.39 ± 0.41, respectively; **P < 0.05). The β-endorphin concentration increased significantly in the experimental group after 2 weeks, but not in the control group

The β-endorphin concentration increased significantly in the experimental group, but not in the control group, after 2 weeks (*P* < 0.05). Neonates in the experimental group had higher β-endorphin levels than those in the control group both at the beginning of hospitalization (1.64 ± 0.39 *vs* 1.18 ± 0.09, respectively; *P* < 0.05) and 2 weeks later (2.29 ± 0.74 *vs* 1.39 ± 0.41, respectively; *P* < 0.05) (Fig. 4).

## Discussion

In this study, we randomly assigned 62 preterm neonates to either an experimental or control group to demonstrate whether CMT can relieve pain such patients in the NICU. Infants in the experimental group underwent daily painful procedures with CMT, while those in the control group underwent daily painful procedures without interference. Details on the daily painful procedures, PIPP scores, and circulatory cortisol and β-endorphin concentrations were analyzed at the beginning of hospitalization and 2 weeks later.

The hospitalized preterm neonates in this study underwent an average of 29.0 to 35.5 painful procedures during 2 weeks of hospitalization. Among these procedures, those related to tracheal aspiration (e.g., tracheal aspiration, nasal aspiration, and chest physiotherapy) and intravenous cannulation (e.g., intravenous cannulation, removal of intravenous lines, and adhesive removal) are the most frequent types of painful procedures performed among preterm infants. This might be because preterm infants commonly have complications such as breathing difficulties and nutrient defects. These complications lead most preterm neonates to require ventilation, support, and prolonged infusion of parenteral nutrition. Thus, the numbers of related procedures are significantly increased.

The PIPP was chosen as the pain measurement tool for this study because it is a composite of seven multidimensional indicators of pain. These pain indicators include physiological, behavioral, and contextual measures that adjust for the influence of gestational age at the time of treatment and the infant’s state of awareness [34–37]. As shown in Fig. 2, after 2 weeks, the PIPP score in the control group had significantly increased and that in the experimental group had significantly decreased; the PIPP score in the experimental group was significantly lower than that in the control group. Therefore, we presume that CMT can reduce the pain response in premature infants.

Cortisol, the steroid end product of the HPA axis, increases with painful procedures [38] and decreases with comforting procedures such as massage [39] and skin-to-skin care [40] in preterm infants. Thus, we supposed that the cortisol concentration would not differ between the two groups at the beginning of hospitalization and would significantly increase in the control group but not in the experimental group after 2 weeks of hospitalization. However, although there was no significant difference in the cortisol concentration between the two groups in this study, the cortisol concentration in the experimental group was slightly lower than that in the control group after a single blood collection at the beginning of hospitalization. After 2 weeks, the cortisol concentration had decreased in both groups, especially in the control group, in which the cortisol concentration had significantly decreased against the beginning of hospitalization. Two meaningful hypotheses can be offered to explain this phenomenon. First, at the beginning of hospitalization, the cortisol level in the control group increased because of a single painful procedure while that in the experimental group decreased because of a single CMT procedure. Measurement of the salivary cortisol concentration may be more accurate because there is no painful procedure at first. This would allow comparison of the baseline concentration and subsequent concentrations after single or repeated pain exposures. Second, after 2 weeks of hospitalization, the lower cortisol concentration may have occurred because repeated exposure to procedural pain was associated with downregulation of the HPA axis, such that the cortisol responses were dampened while the infants were still in the hospital [41]. Overall, the HPA axis responsiveness, cortisol regulation, and normal cortisol concentrations in preterm infants are very complex, and continued research is needed.

In this study, at the beginning of hospitalization, we found that the β-endorphin concentration significantly increased in the preterm infants of the experimental group after a single CMT; this prompted the β-endorphin concentration to change immediately after the intervention. However, no significant differences in the PIPP scores were detected at the beginning. After 2 weeks, the β-endorphin concentration in the experimental group had significantly increased while that in the experimental group had significantly decreased. These data suggest that in preterm infants, although the β-endorphin concentration increased, the increase was not adequate to reduce the pain response after a single intervention; repeating CMT might decrease the pain response by improving the β-endorphin concentration.

This study has shown that CMT is effective in comforting late preterm infants when they undergo painful procedures. As advocates for premature infants, neonatal nurses must continually explore treatment modalities to provide these infants with quality care and hope for a bright future. CMT plays a potential role in this field. A limitation of this study is that it was a pilot study with a small sample size; it would be advantageous to enlarge the sample size. Furthermore, it was a single-center case series that may not represent the situation in China as a whole. Thus, future studies involving other NICUs are needed to map the epidemiology of neonatal pain in China.

## Conclusions

CMT might decrease the pain response of preterm neonates by improving the β-endorphin concentration, but not the blood cortisol concentration.

## Abbreviations

CMT: Combined music and touch intervention
HPA axis: Hypothalamic-pituitary-adrenal axis
NICU: Neonatal intensive care unit
PIPP: Premature Infant Pain Profile
